# Mosquitoes from Europe Are Able to Transmit Snowshoe Hare Virus

**DOI:** 10.3390/v16020222

**Published:** 2024-01-31

**Authors:** Stephanie Jansen, Patrick Höller, Michelle Helms, Unchana Lange, Norbert Becker, Jonas Schmidt-Chanasit, Renke Lühken, Anna Heitmann

**Affiliations:** 1 Faculty of Mathematics, Informatics and Natural Sciences, University of Hamburg, 20148 Hamburg, Germany; stephanie.jansen@uni-hamburg.de (S.J.); schmidt-chanasit@bnitm.de (J.S.-C.); 2 Bernhard Nocht Institute for Tropical Medicine, 20359 Hamburg, Germany; patrick.hoeller@bnitm.de (P.H.); helms@bnitm.de (M.H.); unchana.lange@bnitm.de (U.L.); luehken@bnitm.de (R.L.); 3 Institute for Dipterology, 67346 Speyer, Germany; norbertbecker@gmx.de; 4 Center for Organismal Sudies (COS), University of Heidelberg, 69120 Heidelberg, Germany

**Keywords:** snowshoe hare virus, vector competence, *Aedes*, *Culex*

## Abstract

Snowshoe hare virus (SSHV) is a zoonotic arthropod-borne virus (arbovirus) circulating in colder areas of the Northern Hemisphere. SSHV is maintained in an enzootic cycle between small mammals and mosquitoes, assumably of the genera *Aedes* and *Culiseta*. Symptoms of SSHV human infection can range from asymptomatic to severe neuroinvasive disease. Studies on SSHV transmission are limited, and there is no information available on whether mosquitoes of the genus *Culex* are able to transmit SSHV. Therefore, we investigated six mosquito species via salivation assay for their vector competence. We demonstrated that SSHV can be transmitted by the abundant European *Culex* species *Cx. pipiens* biotype pipiens, *Cx. pipiens* biotype molestus, and *Cx. torrentium* with low transmission efficiency between 3.33% and 6.67%. Additionally, the invasive species *Ae. albopictus* can also transmit SSHV with a low transmission efficiency of 3.33%. Our results suggest that local transmission of SSHV after introduction to Europe seems to be possible from a vector perspective.

## 1. Introduction

Arthropod-borne viruses (arboviruses) have been an increasingly emerging global health threat over the last decades, as the recent epidemics of dengue and chikungunya have shown. The risk of arbovirus transmission increases due to factors such as climate change, environmental changes, and increased traveling and trading, which contributes to the spread of both, invasive mosquito species and the arboviruses themselves [1,2].

Snowshoe hare virus (SSHV) belongs to the *Orthobunyavirus* genus in the *Peribunyaviridae* family within the *Bunyavirales* order, which forms the largest genus of arboviruses worldwide [3]. *Orthobunyavirus* virions are enveloped and have a single-stranded negative-sense tripartite genome. All members of the *Orthobunyavirus* genus are transmitted by arthropods, especially mosquitoes [3]. Based on their serological and genetical relationship, the genus of *Orthobunyavirus* is subdivided into different groups/complexes. The California serogroup (CSG) with SSHV has currently 18 members. The prototype virus of the CSG is the California Encephalitis virus (CEV), isolated from different mosquito pools in the 1940s in the San Joaquin Valley, California (US) [4,5]. In 1952, the first human case of encephalitis caused by the California Encephalitis virus was described [6]. The first isolation of SSHV took place in 1959 in Bitterroot Valley, Montana (US) from the blood of a *Lepus americanus,* the snowshoe hare [7]. This was the first time a CSG member was isolated from a vertebrate and not from mosquitoes.

Orthobunyaviruses are found across various regions, ranging from tropic to arctic areas on all continents, with the exception of Antarctica [8]. SSHV is distributed in colder regions in the Northern Hemisphere, i.e., Canada, USA, and Russia [8,9]. The clinical course of SSHV infection in humans can range from asymptomatic to mild illnesses to severe neuroinvasive diseases. In fact, in the US, mainly three members of the CSG are causing neuroinvasive diseases: La Crosse Virus (LACV), Jamestown Canyon Virus (JCV), and SSHV [10]. While LACV and SSHV affect mainly children, JCV affects primarily adults, and the reason for this is still unknown [11]. There are only a few reports of SSHV infection in humans from the 1970s and 1980s from various Canadian provinces, one pediatric neuroinvasive case from Novia Scotia in 2006, and one case of meningoencephalitis from Manitoba in 2016 [12,13,14,15,16,17]. Serological studies conducted in Alaska (US), an endemic area of SSHV, revealed antibody positivity rates of 42% in the 1980s and 6.8% in the 1990s, indicating a significant number of undetected human cases [18,19].

SSHV circulation is sustained through an enzootic cycle involving mosquitoes and mammals. It is assumed that the primary and amplifying hosts are small mammals. For instance, serological studies conducted in Alaska and Wyoming (US), as well as in Newfoundland (Canada), have indicated high seroprevalences in snowshoe hares [18,20,21]. As SSHV-positive mosquitoes have been detected northwards of the distribution area of snowshoe hares, other mammalian species must be involved in the transmission cycle [22]. In Montana (US), ground squirrels have tested positive for SSHV antibodies, while voles, chipmunks, rats, and other small mammals in this area were negative [23]. Hares, rabbits, lemmings, and red-backed voles have been found SSHV-antibody-positive in Alaska (US) [24]. Additionally, larger wild animals such as bison, dall sheep, bovines, sheep, deer, and moose have shown positive serological results [18,20,25,26]. Laboratory studies on small mammals revealed snowshoe hares, squirrels, rats, and voles susceptible to SSHV, but marmots and the white-footed mouse do not develop viremia [23]. However, laboratory studies have provided no evidence of SSHV infection in larger mammals, and the experimental infection of deer, elk calves, and dogs failed [23,27]. Similar to several arboviruses, e.g., West Nile virus, among larger mammals, horses appear uniquely susceptible to SSHV, potentially developing encephalitis [28,29,30]. Serological studies in Newfoundland (Canada) have shown a low seroprevalence in horses, suggesting SSHV infection of horses is predominantly asymptomatic; only a small number of horses develop encephalitis, but spontaneous recovery is possible [20,29].

Field studies identified several mosquito species carrying SSHV, but this does not necessarily confirm them as competent vectors. Various *Aedes* species, including *Ae. fitchii*, *Ae. canadensis*, *Ae. communis*, *Ae. cinereus*, *Ae. hexodontus* complex, *Ae. punctor* complex, or *Ae. vexans* have tested positive for SSHV [23,31,32,33,34,35,36]. Additionally, SSHV-positive *Culiseta* species were also collected, e.g., *Cs. impatiens* and *Cs. inornata* [23,34]. Notably, there are no reports of SSHV in *Anopheles* or *Culex* mosquitoes, even though these have been analyzed in studies [34,37]. This resulted in the suggestion that SSHV is a “non-*Culex*” virus, and the current distribution of SSHV aligns with the high abundance of *Aedes* and *Culiseta* mosquitoes [38]. However, further research is needed to confirm this hypothesis. 

SSHV is found in regions with long harsh winters and short summers. LeDuc et al. proposed that the virus winter maintenance could occur through transovarial transmission, persistence in infected vertebrates, or overwintering in mosquitoes [39]. In support of the transovarial transmission theory, SSHV-positive *Aedes* larvae have been collected in the field, indicating this as a method for SSHV overwintering [36,40]. Another plausible mechanism is the overwintering of infected adult mosquitoes. *Culiseta* mosquitoes are susceptible to SSHV by intrathoracic injection and able to transmit SSHV, even after incubation at temperatures as low as 0 °C or 13 °C [41,42]. SSHV maintenance in vertebrates is also possible, as SSHV could be detected in the mosquito-free season in several small mammals, like hares and voles [24]. However, there are limited laboratory studies on the vector competence of different mosquito species for SSHV. Transmission of SSHV to suckling mice was shown for *Ae. Provocans*, *Ae. Abserratus-punctori*, and *Ae. triseratus* at 19 °C/23 °C [43,44]. Replication of SSHV was also detected in intrathoracic-injected *Ae. Canadensis*, as well as *Cs. iornata* mosquitoes incubated at temperatures from 13 °C to 24 °C [45]. *Aedes aegypti* and *Cs. iornata* showed a vector competence even at low temperatures: 13 °C/24 °C for *Ae. aegypti* and 13 °C for *Cs. iornata* [45]. Further studies with *Cs. iornata* incubated at temperatures around the freezing point showed positive specimens even after 194 days [45]. 

Three members of the CSG are distributed in Europe: Tahyna virus (TAHV) on the whole continent, as well as Inkoo virus (INKV) and Chatanga virus (CHATV), which both only occur in the northern areas of Europe. While it is assumed that transmission of TAHV takes place by a broad range of mosquito species (*Aedes*, *Culex*, and *Culiseta*), the transmission of INKV and CHATV probably only occurs by *Aedes* mosquitoes, but this assumption is mainly based on virus detection in field-caught mosquitoes, which again does not necessarily confirm them as competent vectors [8]. Given the recent emergence of certain orthobunyaviruses with public and veterinary health relevance in new areas, such as the appearance of the Cache Valley virus in New York, it is crucial to possess basic knowledge about these viruses to prevent larger outbreaks/epidemics [46,47]. To address the substantial knowledge gap regarding the vector competence of mosquitoes for SSHV, we conducted this study, with a special focus on *Culex* species. This information will help to estimate the risk of SSHV transmission in currently nonendemic areas such as Europe.

## 2. Materials and Methods

Egg rafts of *Culex pipiens* s.s./*Culex torrentium* were collected in the field during the summer of 2023 in northern Germany (Lon: 53.467821/Lat: 9.831346). Larvae were reared at room temperature with a 12:12 light:dark photoperiod. Species identification as *Culex pipiens* biotype pipiens (*Cx. pipiens* pipiens) and *Cx. torrentium* was performed by extracting DNA of a pool of 5 L1/L2 larvae per egg raft (DNeasy blood & tissue kit, Qiagen, Hilden, Germany) and multiplex quantitative real-time PCR (qPCR) as described by Rudolf et al. (HotStarTaq master mix kit, Qiagen, Hilden, Germany) [48]. Pupae were placed in an insectary with a relative humidity of 70%, 26 °C, and a 12:12 light:dark photoperiod, including 30 min twilight. To exclude natural arbovirus infections, 10 randomly selected adult mosquitoes per species were tested by pan-Orthobunya-, pan-Flavivirus-, and pan-Alphavirus-PCR, confirming all specimens as negative [49,50,51]. Lab strains of *Culex pipiens* biotype molestus (*Cx. pipiens* molestus) (established since 2011 from egg rafts collected in Heidelberg, Germany), *Culex quinquefasciatus, Aedes aegypti* (both long-established colonies from Bayer, Leverkusen, Germany), and *Aedes albopictus* (established with eggs from the field in Heidelberg in 2016/2017) were reared in the insectary likewise.

Female mosquitoes with an age of 4–14 days were starved for 24 (*Aedes*) or 48 (*Culex*) hours. An artificial blood meal was performed at 24 °C for two hours, containing 50% human blood (expired blood preservation), 30% of an 8% fructose solution, 10% filtrated bovine serum (FBS), and 10% virus stock, final virus concentration was 2.2 × 10^6^ FFU/mL. SSHV stock was propagated on BHK-21 cells (*Mesocricetus auratus*, CCVL L 0179, Friedrich-Loeffler-Institute, Riems, Germany), using the SSHV strain ATTC VR-711 [52]. A blood meal was offered either via cotton stick for all *Culex* mosquitoes, reaching a feeding rate (FR, number of engorged females per number of fed females) of 67.1% for *Cx. pipiens* pipiens, 55.7% for *Cx. pipiens* molestus, 50.0% for *Cx. torrentium* and 95.5% for *Cx. quinquefasciatus* or via two 50µL drops for *Aedes* mosquitoes, reaching an FR of 42.6% for *Ae. aegypti* and 81.0% for *Ae. albopictus* (Table 1). Only fully engorged females were used for the experiments. Mosquitoes were incubated for 14 days at 70% humidity and fluctuating temperature profiles of 18 °C or 24 °C with variations of +/−5 °C within 24 h. The highest temperature was reached in the middle of the light period, and the lowest temperature was reached in the middle of the dark period to mimic day and night fluctuation. Fructose was offered continuously via cotton pads and refreshed every 2–3 days. The survival rate (SR, number of alive mosquitoes 14 days post infection (dpi) per number of fed females) of all mosquito species was in a range of 68–100% (Table 1). All experiments were performed in 2 replicates.

Vector competence was analyzed at 14 dpi via salivation assay as previously described [53]. Briefly, mosquitoes were anesthetized with CO_2_ to remove legs and wings. The proboscis was put into a 10 µL tip containing 10 µL of phosphate-buffered saline (PBS) and incubated for 30 min. PBS-saliva solution was pipetted onto BHK cells in a 96-well plate to observe if the saliva contained infectious virus particles, which would induce a cytopathic effect during the next 7 days. If CPE was observed, RNA of the supernatant was extracted (QIAmp viral RNA mini kit, Qiagen, Hilden, Germany) and tested via qRT-PCR (QuantiTect Reverse Transcription kit, Qiagen, Hilden, Germany) using the Primers SHL80C (CAACAATTCTTAGCTAGGATTAA) and SHL146V (GATCGACATCTATATCTTTGGCA) located in the L-segment [54], with an addition of the VetMAX^TM^ Xeno^TM^ Internal Positive Control (Applied Biosystems, Thermo Fisher Scientific Corporation, Waltham, MA, USA). A series of 1.19 × 10^3^, 1.19 × 10^4^, 1.19 × 10^5^ dilutions of a synthetic SSHV standard (5′-ATGCAACAATTCCTAGCTAGGATTAATGCTGCAAGAGATGCATGTGTTGCCAAAGATATAGATGTCGATCCTA-‘3) was used as a positive control.

The transmission rate (TR, number of SSHV-positive saliva per number of SSHV-positive bodies) and transmission efficiency (TE, number of SSHV-positive saliva per fed females) were calculated. To estimate the amount of infectious virus particles, saliva was titrated as described by Jansen et al. [55]. The RNA of mosquito bodies, excluding legs and wings, was extracted (MagMAX CORE nucleic acid purification kit, Applied Biosystems, Thermo Fisher Scientific Corporation, Waltham, MA, USA), and RT-qPCR was performed as mentioned above. Infection rate (IR, number of SSHV-positive bodies per fed females) and mean body titer of each specimen were calculated using a series dilution of the above-mentioned SSHV standard.

The RT-qPCR was validated in accordance with the “Minimum Information for Publication of Quantitative Real-Time PCR Experiments” guidelines as outlined by Bustin et al. [56]. A series of ten-fold dilutions from 1.19 to 1.19 × 10^9^ copies/µL of the SSHV standard were analyzed in five replicates according to the above-mentioned RT-qPCR protocol. The limit of detection was determined to be 2.68 × 10^5^ copies/mosquito body, and the standard deviation of C_q_s at this concentration was 1.173. The linear dynamic range was established between 2.68 × 10^5^ and a minimum of 2.68 × 10^11^ copies/mosquito body, meaning that the concentration is in a linear proportion to the PCR signal in this range and can therefore be considered reliable. From the calibration curves, the coefficient of determination was calculated to be 0.9968. The slope was −3.539, while the y-intercept was at 53.304. Finally, the PCR efficiency was 0.9184, i.e., 91.8% of the target molecules were amplified in each step.

## 3. Results

All investigated mosquito species were susceptible to SSHV and four of the six were capable of transmitting SSHV, i.e., *Ae. albopictus*, *Cx. pipiens* pipiens, *Cx. pipiens* molestus, and *Cx. Torrentium* (Table 2). No positive saliva was detected for *Ae. Aegypti* and *Cx. Quinquefasciatus*.

*Aedes aegypti* showed the lowest IR of all species, with an IR of 0.0% at 18 °C +/− 5 °C and 10.0% at 24 °C +/− 5 °C (Table 2). Both temperature profiles are presented as 18 °C and 24 °C, respectively, in the following results and discussion. Likewise, the mean body titer of 3.6 log10 copies per specimen was the lowest titer of all species, resulting in no transmission. In contrast, *Ae. albopictus* showed the overall highest IRs of 96.7% at 24 °C and 50.0% at 18 °C. Moreover, the body titers of 7.0 log10 copies per specimen at both temperature profiles are the highest values over all species. While the TR is higher at 18 °C with 6.7% in comparison with 3.5% at 24 °C, the TE with 3.3% is identical for both temperatures.

For *Cx. pipiens* pipiens, the IR was 23.3% at 18 °C and 26.7% at 24 °C, with mean body titers of 4.7 and 5.4 log10 copies per specimen, respectively. Transmission was only observed at 24 °C with a TR of 12.5% and a TE of 3.3%. *Culex pipiens* molestus showed lower IRs, with 16.7% at 18 °C and 3.3% at 24 °C. The titer was slightly lower at the higher temperature of 24 °C with 4.8 log10 copies per mosquito compared with 6.0 log10 copies per mosquito at 18 °C. Transmission only took place at 18 °C, with a TR of 20.0% and a TE of 3.3%. *Culex torrentium* had the highest IR of all investigated *Culex* species, with 50.0% at 18 °C and 40.0% at 24 °C. The body titer was higher at the higher temperature, with 5.8 log10 copies per specimen in comparison with 5.0 log10 copies per specimen at the lower temperature. Transmission was only observed at the higher temperature with a TR of 16.67% and a TE of 6.67%, which is the highest measured TE for all species. No transmission was observed for *Cx. quinquefasciatus*, but infection was detected at both temperatures with an IR of 3.3% with a body titer of 6.1 log10 copies per specimen at 18 °C and an IR of 43.3%, with a body titer of 5.1 log10 copies per specimen at 24 °C.

In all detected saliva samples, a cytopathic effect was only present in the first well of the saliva dilution, which resulted in a concentration of <10 infectious virus particles per saliva sample.

## 4. Discussion

In this study, we found that *Culex pipiens* pipiens, *Cx. pipiens* molestus, and *Cx. torrentium* are capable of transmitting SSHV. To the best of our knowledge, this is the first demonstration of SSHV transmission by *Culex* mosquitoes. Moreover, this is the first demonstration of SSHV transmission by the highly invasive mosquito species *Ae. albopictus*. Although no positive saliva was detected in *Ae. aegypti* and *Cx. quinquefasciatus*, both species were susceptible to SSHV infection. The TE of SSHV by both *Aedes* and *Culex* genera was relatively low at around 3.3%, with a slightly higher rate of 6.7% observed for *Cx. torrentium*. This contrasts with other members of the CSG, which show a clear difference in transmission by the different mosquito genera. For example, transmission of the closely related LACV [57] has been demonstrated in *Culex*, specifically *Cx. restuans* and *Cx. pipiens*, with TE values also ranging under 10% [58]. However, vector competence studies with LACV and *Ae. triseriatus* showed significantly different results, yielding high TE values of 40% [58]. Another example of a CSG member is TAHV, for which recent studies with a newly discovered TAHV strain from China showed infection of *Ae. albopictus* and *Cx. pipiens pallens*, but SSHV-positive saliva was only detected in *Ae. albopictus* [59]. Other members of the CSG group, like CEV and JCV, are also known to be transmitted by several *Aedes* species, with transmission rates ranging from high to low levels [60,61]. The transmission of JCV by *Ae. albopictus* was recently demonstrated with TRs ranging from 13% to 30% (7 dpi, incubation temperature of 27 °C) [62]. The hypothesis that SSHV is a “non-*Culex*” virus and that the current distribution of SSHV corresponds with the high abundance of *Aedes* and *Culiseta* mosquitoes is probably not correct [38]. Risk assessment for SSHV in endemic areas may need to be reconsidered, as *Culex* mosquitoes as vectors need to be taken into account.

In this study, transmission was shown at 24 °C for three species (*Ae. albopictus*, *Cx. pipiens* pipiens, *Cx. torrentium*) and at 18 °C for two species (*Ae. albopictus*, *Cx. pipiens* molestus). Therefore, it seems that the transmission of SSHV is not limited by lower temperatures. This aligns with the observations of McLean et al., who detected SSHV transmission by *Ae. aegypti* and *Cs. Iornata* at incubation temperatures of 24 °C and 13 °C [45]. Abundant European *Culex* mosquito species, as well as the invasive *Ae. Albopictus*, are susceptible and able to transmit SSHV under the prevailing temperatures in Europe. Thus, travel-associated SSHV introduction via mosquitoes/humans or introduction by infected vertebrate hosts, migrating from Russia to Northern Europe, is a potential scenario. The theories of LeDuc et al. for winter maintenance of SSHV are also conceivable for Europe [39]. Transovarial transmission, a known mechanism for several CSG viruses in *Aedes* mosquitoes, appears feasible for SSHV in European *Aedes* species [63]. The investigated *Culex* mosquitoes from Germany, which overwinter as adults similar to *Culiseta* mosquitoes from Northern America, could also facilitate SSHV maintenance [64,65,66]. Whether SSHV can establish an enzootic cycle in Europe would also depend on the hosts. However, due to the broad host range of different species of small mammals [23], it can be assumed that European hares, squirrels, or other small mammals could serve as amplifying hosts. However, these experimental studies are missing.

Another factor that should be kept in mind is the risk of reassortment of viral genome segments, common with orthobunyaviruses [67]. If genetically and antigenic close orthobunyaviruses infect the same cell, an exchange of the three segments can take place, leading to a reassortment of segments and the emergence of novel viruses. The reassortment of orthobunyaviruses in the laboratory was first described for LACV in dually infected mosquitoes [68]. The phenotypes of reassortment viruses can vary significantly from their parental strains, with either increased or decreased virulence. For example, the laboratory reassortment of LACV and JCV shows a loss of pathogenicity in mammals [69]. An example of the opposite effect is the natural reassortment of the Ngari virus (NRIV), containing the L- and S-segment of the Bunyamwera virus (BUNV) and the M-segment of the Batai virus (BATV), both belonging to the bunyamwera serogroup of the orthobunyaviruses [70]. NRIV shows a higher pathogenicity for mammals in comparison with the parental viruses, i.e., BUNV and BATV infections in humans are mainly asymptomatic or associated with febrile illness, while NRIV causes hemorrhagic fever [71]. Interestingly, laboratory studies with an NRIV-like virus showed an increased pathogenicity in mammalian cells in comparison with the parental viruses BUNV and BATV but a decreased growth in insect cells [72]. Therefore, the assessment of new reassortment viruses must always take the host as well as the vector into account in order to make a risk assessment. The observations of Beaty et al. on the reassortment viruses of LACV and SSHV revealed an important function for the M-segment: encoding for the glycoproteins and the nonstructural proteins NSm. Reassortment viruses containing the M-segment of LACV were efficiently transmitted by *Ae. triseratus*. In contrast, reassortment viruses containing the M-segment of SSHV were only inefficiently transmitted [73,74,75]. However, there are only a few states in the US where LACV and SSHV occur together and natural reassortment could take place, but so far, there have been no reports from the field. Two members of the CSG are distributed in Northern Europe, INKV and CHATV, whereby CHATV is phylogenetically most closely related to SSHV within the CSG, and the reassortment with SSHV in coinfected hosts/vectors is quite conceivable [8,57]. In fact, SSHV occurs in Russia, where INKV, CHATV, and TAHV co-circulate. A sequence analysis of several isolates in Russia belonging to the CSG showed potential SSHV reassortments [9]. With expanding mosquito populations and the presence of other CSG members in Northern Europe and Russia, the risk of novel SSHV reassortments increases.

## 5. Conclusions

SSHV can be transmitted by abundant, native *Culex* mosquitoes in Europe with low TE: *Cx. pipiens* biotype pipiens, *Cx. pipiens* biotype molestus, and *Cx. torrentium.* Additionally, the invasive species *Ae. albopictus* can also transmit SSHV with low TE. Both investigated tropical mosquito species, *Ae. aegypti* and *Cx. quinquefasciatus,* were not able to transmit SSHV. Considering these findings, the introduction of SSHV to Europe appears feasible from the vector as well as the climate perspective. However, further investigation of the potential vertebrate hosts and introduction pathways is needed.

## Figures and Tables

**Table 1:** Number of mosquito specimens per experiment condition (total input), calculation of feeding rate (FR, number of engorged females per number of fed females), and survival rate (SR, number of alive mosquitoes 14 days post infection per number of fed females) for infection experiments with snowshoe hare virus 14 days post infection.

Species	Temperature (°C)	Total Input	FR (%)	SR (%)
*Aedes aegypti*	18° +/− 5 °C	96	60.4 (58/96)	69.0 (40/58)
	24° +/− 5 °C	122	42.6 (52/122)	80.8 (42/52)
*Aedes albopictus*	18° +/− 5 °C	60	83.3 (50/60)	68.0 (34/50)
	24° +/− 5 °C	84	81.0 (68/84)	76.5 (52/68)
*Culex pipiens* biotype pipiens	18° +/− 5 °C	83	53.0 (44/83)	77.3 (34/44)
	24° +/− 5 °C	97	55.7 (54/97)	87.0 (47/54)
*Culex pipiens* biotype molestus	18° +/− 5 °C	115	44.4 (51/115)	96.1 (49/51)
	24° +/− 5 °C	79	67.1 (53/79)	94.3 (50/53)
*Culex torrentium*	18° +/− 5 °C	56	96.4 (54/56)	87.0 (47/54)
	24° +/− 5 °C	56	94.6 (53/56)	94.3 (50/53)
*Culex quinquefasciatus*	18° +/− 5 °C	75	53.3 (40/75)	100.0 (40/40)
	24° +/− 5 °C	90	46.7 (42/90)	97.6 (41/42)

**Table 2:** Calculation of infection rate (IR, number of SSHV-positive bodies per fed females), mean body titer, transmission rate (TR, number of SSHV-positive saliva per number of SSHV-positive bodies), and transmission efficiency (TE, number of SSHV-positive saliva per fed females) for infection with snowshoe hare virus 14 days post infection; thirty specimens were investigated per condition (n.a. = not applicable for the mean if there were no positive specimens or for the confidence interval if there was only one positive body).

Species	Temperature (°C)	IR (%)	Mean Body Titre log10 Copies/Mosquito Specimen (95% Confidence Interval)	TR (%)	TE (%)
*Aedes aegypti*	18° +/− 5 °C	0.0 (0/30)	n.a.	0.0 (0/0)	0.0 (0/0)
	24° +/− 5 °C	10.0 (3/30)	3.6 (1.8–5.4)	0.0 (0/0)	0.0 (0/0)
*Aedes albopictus*	18° +/− 5 °C	50.0 (15/30)	7.0 (6.2–7.8)	6.7 (1/15)	3.3 (1/30)
	24° +/− 5 °C	96.7 (29/30)	7.0 (6.4–7.6)	3.5 (1/29)	3.3 (1/30)
*Culex pipiens* biotype pipiens	18° +/− 5 °C	23.3 (7/30)	4.7 (4.1–5.2)	0.0 (0/0)	0.0 (0/0)
	24° +/− 5 °C	26.7 (8/30)	5.4 (4.2–6.7)	12.5 (1/8)	3.3 (1/30)
*Culex pipiens* biotype molestus	18° +/− 5 °C	16.7 (5/30)	6.0 (3.9–8.2)	20.0 (1/5)	3.3 (1/30)
	24° +/− 5 °C	3.3 (1/30)	4.8 (n.a.)	0.0 (0/0)	0.0 (0/0)
*Culex torrentium*	18° +/− 5 °C	50.0 (15/30)	5.0 (4.7–5.4)	0.0 (0/0)	0.0 (0/0)
	24° +/− 5 °C	40.0 (12/30)	5.8 (5.0–6.6)	16.7 (2/12)	6.7 (2/30)
*Culex quinquefasciatus*	18° +/− 5 °C	3.3 (1/30)	6.1 (n.a.)	0.0 (0/0)	0.0 (0/0)
	24° +/− 5 °C	43.3 (13/30)	5.1 (4.8–5.5)	0.0 (0/0)	0.0 (0/0)

## Data Availability

The data presented in this study are available on request from the corresponding author.
